# Correlation Between Color and Bubble Microstructural Characteristics in Baltic Amber

**DOI:** 10.3390/ma19101978

**Published:** 2026-05-11

**Authors:** Yue Luo, Xiangyu Zhang, Guanghai Shi

**Affiliations:** 1 School of Art and Design, Wuhan University of Technology, Wuhan 430070, China; yuezi1219@gmail.com; 2 State Key Laboratory of Geological Processes and Mineral Resources, China University of Geosciences, Beijing 100083, China; 3057230005@email.cugb.edu.cn

**Keywords:** Baltic amber, color, beeswax-amber, bubbles, SEM, Mie scattering, Rayleigh scattering

## Abstract

**Highlights:**

**What are the main findings?**

- Baltic amber color correlates with internal bubble size, number, and density.
- White beeswax-amber contains abundant micro- to nanoscale bubbles.
- Beeswax-amber’s hue is contingent upon whether selective absorption or light scattering prevails. The disparity between these two visual systems yields unique macroscopic colors.
- Rayleigh and Mie scattering may jointly affect white amber spectral contributions.

**What are the implications of the main findings?**

- Reveals the microstructural origin of color variation in the Baltic amber.
- Demonstrates the role of light scattering in amber coloration.
- Provides insight into structure–optical property relationships in amber.

**Abstract:**

Baltic amber exhibits a wide range of colors and has attracted considerable attention in materials science. Previous studies have mainly focused on the origin and formation characteristics of beeswax-amber, while the relationship between beeswax-amber color and the microstructural characteristics of internal bubbles remains poorly understood. Ten beeswax-amber specimens exhibiting a color gradient from yellow to white were selected. Scanning electron microscopy (SEM) was used to examine and analyze their internal structures, with a focus on documenting bubble size, number, and density characteristics. Ultraviolet (UV) illumination was employed for qualitative optical observation, and Fourier-transform infrared (FTIR) spectroscopy was conducted to identify component phase and spectra. The correlation between bubbles and color was analyzed to infer the origin of white beeswax-amber’s coloration and explore the mechanisms underlying beeswax-amber’s color variation. Results indicate that beeswax-amber coloration is closely linked to its microscopic bubble characteristics. The microstructure satisfies conditions for Mie scattering, some white beeswax-amber samples contain abundant nanoscale bubbles, triggering a combined effect of Rayleigh and Mie scattering. These results demonstrate that the color of Baltic amber is governed not only by its intrinsic body color but also by the synergistic optical effects arising from internal bubble microstructures, providing a physically grounded explanation for its diverse appearances.

## 1. Introduction

Amber is a kind of gems spectral contribution that comes from the resin of ancient trees. This resin hardens when it meets sunlight and air. The amber then becomes hard gets buried under layers of soil and rocks and over time it turns into a fossil [1]. Amber from different places looks different because it is made from different tree resins and gets preserved in different ways. For example, amber from Burma has lots of living things trapped inside it and it has very detailed patterns that you can only see with a microscope [2]. Baltic amber represents the most extensive known deposit of fossilized plant resin on the planet and has been harvested, exchanged, and examined for over five thousand years. This copolymer consists of labdanoid diterpenes, primarily communic acid and communol, which include succinic acid as a cross-linking agent, with total succinate concentrations varying from 1% to 8% by mass, a characteristic chemical signature that differentiates it from all other significant fossil resin deposits [3].

The commercial and cultural importance of Baltic amber is considerable. It has served as a gemstone, a carving medium, and a constituent in traditional medicine across several civilizations, and it continues to be one of the principal gem materials in the jewelry industry of the Baltic region. In this sector, beeswax-amber, particularly the opaque to translucent form, garners specific market interest due to its extensive color distribution. It is often classified by color, such as pork fat amber, chicken fat yellow, white amber, gray amber, blood amber, brown amber, and green amber. Some types are named based on the distribution of transparent amber and beeswax-amber within them, like golden sand amber, golden twisted amber, golden floating amber, and cave amber. The beeswax-amber portions in the latter group are still predominantly described by color. Beeswax-amber primarily originates from Baltic coastal nations and regions like Ukraine, exhibiting a rich spectrum of hues—typically pale yellow, yellow, bright yellow, orange-yellow, brownish-yellow, and white—encompassing nearly all color and appearance characteristics of beeswax-amber. The coloration of beeswax-amber results from multiple factors.

The scientific literature has examined the color of beeswax amber from several perspectives, although has not properly synthesized them. The molecular composition has historically been seen as a primary factor; the amber spectral contributions, within the yellow-to-brown spectrum, is predominantly linked to the carbonyl chromophore. During the diagenetic process, the resin undergoes polymerization and oxidation reactions. Because beeswax-amber molecules have distinct interior structures, they absorb visible light in diverse ways. This is the source of beeswax-amber’s base spectral contributions, which might be orange or yellow. If beeswax-amber gets buried for a time or is out in the weather, its hue might change. This occurs when external objects enter the beeswax-amber and when the beeswax-amber’s surface oxidizes [4].

Initial investigations of beeswax-amber predominantly concentrated on its chemical makeup and production processes [5]. As research advanced, academics increasingly acknowledged that beeswax-amber’s opacity and unique coloration are influenced not only by its chemical composition and oxidation processes but also by its interior structural attributes [6]. Contemporary beeswax-amber study predominantly utilizes spectrum analysis and fluorescence characteristics to differentiate natural beeswax-amber from imitations, ascertain origin and types, and delineate its features and quality [7]. However, research on molecular spectroscopy infrequently quantifies microstructure, whereas microstructural analyses seldom confirm the chemical equivalence of the samples under comparison [8]. Research utilizing microscopic analysis reveals variations in bubble morphology in beeswax-amber from different sources. Bubbles influence the clarity and quality of amber, however related findings mostly pertain to the causes and mechanisms of development [9]. Moreover, current research generally focuses on individual specimens instead of exploring internal feature changes among numerous samples displaying continuous color shifts. The quantitative correlations between structural properties and color variations are insufficiently investigated, and comprehensive analyses of beeswax-amber’s color formation are absent. Studies on the microscopic characteristics of Baltic amber have revealed a significant correlation between internal bubbles and coloration [10]. This article carefully examines the size, number, and density of bubbles in beeswax-amber displaying changes in color sequence.

This study directly tackles the issue by selecting ten beeswax amber specimens that exhibit a consistent color gradation from yellow to white, and analyzing them using scanning electron microscopy and Fourier-transform infrared spectroscopy. SEM characterization records bubble size, number density, and area percentage for all samples, whilst FTIR analysis examines if the color fluctuation correlates with any systematic alteration in molecular composition. Additionally, it aims to investigate alternative color-forming mechanisms in Baltic amber.

## 2. Materials and Methods

### 2.1. Sample Selection and Macroscopic Characterization

**Figure 1:**
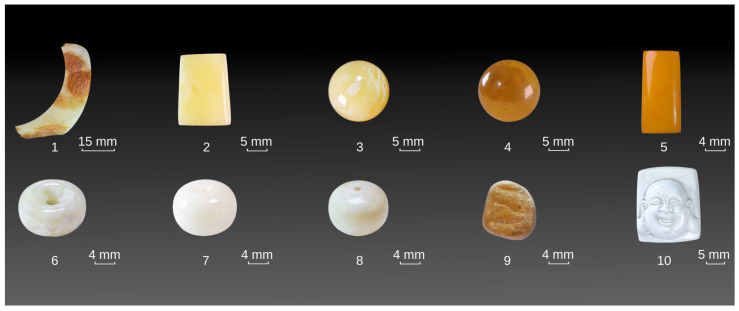
Baltic amber samples with sample No. 1~10.

All research specimens were sourced from Kaliningrad, Russia. The Baltic amber samples were sourced from commercially available materials, encompassing both unrefined specimens and processed products (e.g., beads and carved artifacts). The samples were chosen according to color variation. Ten experimental specimens of diverse colors and luminance were chosen (Figure 1). Employing the Munsell Color Book—Glossy Edition [11] to categorize the beeswax-amber’s body color by hue, value, and chroma. The body color of the beeswax-amber was denoted using the format: lightness-chroma level + hue category. The samples were selected based on color variation, while differences in shape reflect natural morphology and do not affect the analysis. Both naturally exposed and lightly polished surfaces were examined, and only regions minimally affected by polishing were selected for analysis. The hues and attributes of the samples are enumerated in Table 1.

### 2.2. Test Method

Internal characteristics of beeswax-amber specimens were examined using scanning electron microscopy (SEM) at the Electron Probe and Electron Microscopy Laboratory of the Institute of Geophysics and Geophysics, Chinese Academy of Sciences. Enlarged images were acquired with a FEI Nova Nano450 field emission scanning electron microscope (FEI Company, Hillsboro, OR, USA), combined with an Oxford Instruments Aztec EBSD system and an energy-dispersive X-ray spectroscopy (EDS) detector (X-MAXN 80, Oxford Instruments, Oxford, UK). Test parameters: Accelerating voltage of 2 kV, field scan resolution of 4k × 4k, and carbon coating applied [12]. For SEM observation, the selected amber samples were analyzed without additional surface modification to preserve their natural microstructure. Infrared spectra were acquired using a BRUKER TENSOR 27 Fourier-transform infrared (FTIR) spectrometer (TENSOR 27, Bruker, Ettlingen, Germany) in specular reflection mode. Each spectrum was collected over the range of 4000–400 cm^−1^ with a spectral resolution of 4 cm^−1^ and 16 scans. The obtained spectra were subjected to baseline correction and Kramers–Kronig (K–K) transformation. Due to minor damage in one specimen (Sample 9), FTIR measurements were successfully conducted on the remaining nine samples. Ultraviolet (UV) illumination was used for qualitative observation. A UV light source (long-wave UV, ~365 nm; short-wave UV, ~254 nm) was used as the excitation source. No quantitative spectral measurements were performed.

## 3. Results

### 3.1. SEM Analysis

**Figure 2:**
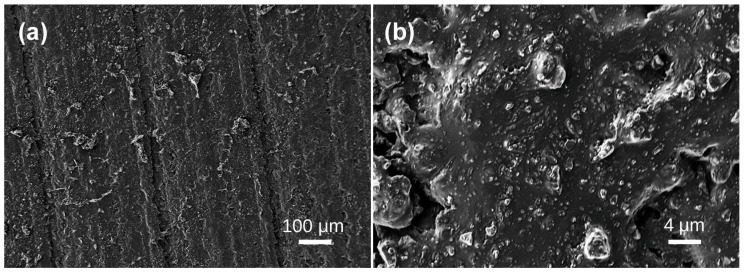
SEM images of Baltic amber sample No. 1: (**a**) low magnification; (**b**) high magnification.

**Figure 3:**
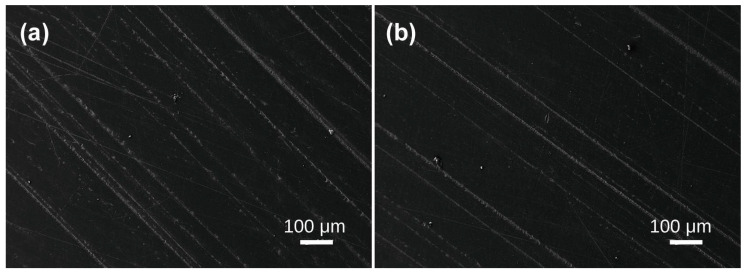
SEM images of Baltic amber sample No. 2 showing no bubbles in different domains (**a**,**b**) at identical magnification.

**Figure 4:**
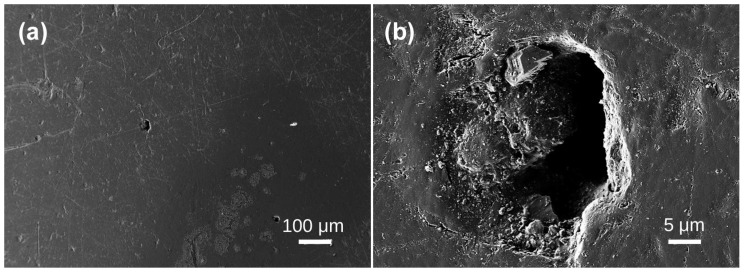
SEM images of Baltic amber sample No. 3: (**a**) low magnification; (**b**) high magnification.

**Figure 5:**
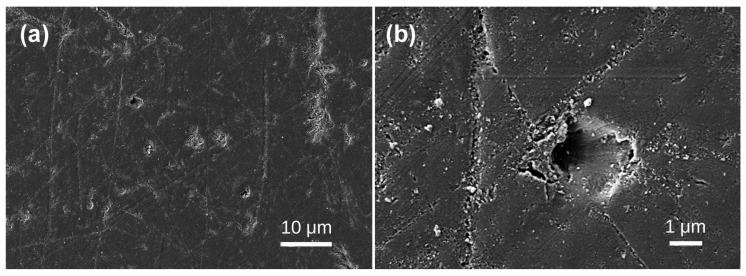
SEM images of Baltic amber sample No. 4: (**a**) low magnification; (**b**) high magnification.

Under scanning electron microscopy, the samples exhibit diverse morphologies, revealing layered structures and various bubble formations. The bubble distribution can be broadly categorized into three scales: extremely sparse distribution in Samples 1, 2, 3, and 4; scattered distribution in Sample 5; and dense distribution in Samples 6, 7, 8, and 10. In Sample 1, lamellar structures are evident (Figure 2a), with interior bubbles being exceedingly rare and challenging to detect. Irregular bubbles are randomly dispersed over the test area, with diameters of approximately 4–5 μm (Figure 2b). No bubbles were seen in any examined region of Sample 2 (Figure 3). Sample 3 had a random distribution of individual bubbles with considerable size variation, with giant bubbles attaining diameter of 15 μm (Figure 4b) and little bubbles measuring merely 1.5 μm in diameter. The examined portion of Sample 4 exhibited a solitary irregular tiny bubble measuring 3 μm in diameter (Figure 5).

**Figure 6:**
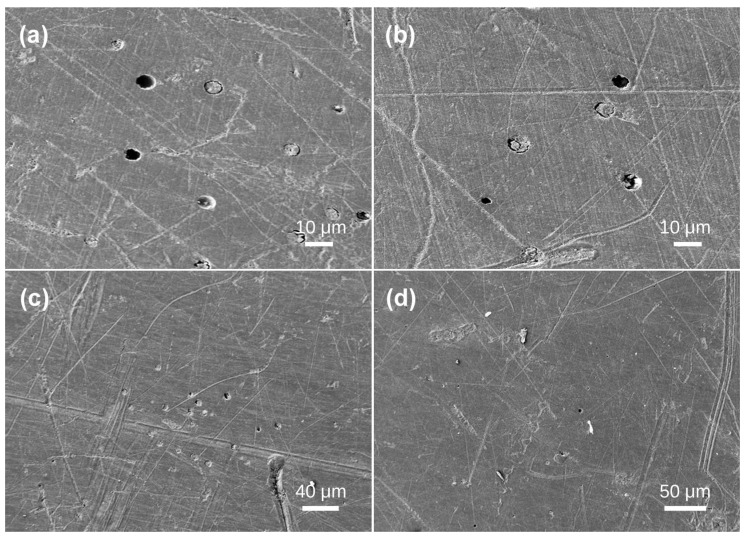
SEM images of Baltic amber sample No. 5: (**a**,**b**) high magnification; (**c**,**d**) low magnification.

Sample 5 was segmented into four sections (a, b, c, d) for analysis. Region a exhibited randomly scattered ellipsoidal holes with sizes around 10 μm (Figure 6a). Region b exhibited heightened bubble density; at 554× magnification, two dimensions of ellipsoidal bubbles were discernible: mostly 10 μm bubbles, accompanied by smaller 5 μm bubbles (Figure 6b). In region c, bubbles were aggregated in a specific location. Like region b, the bubbles displayed two diameters: 10 μm and 5 μm (Figure 6c). Region D had a diminished number of bubbles relative to the first three sections. Only two bubbles were identified, one measuring 5 μm in diameter and the other 8 μm (Figure 6d).

**Figure 7:**
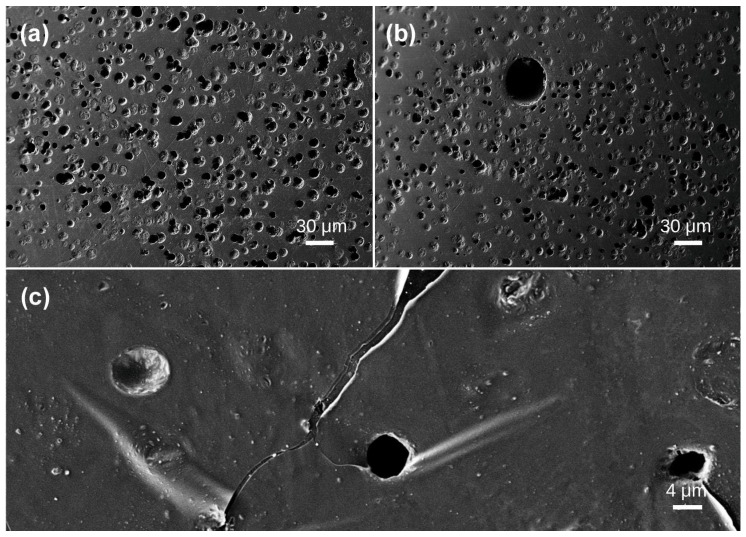
SEM images of Baltic amber sample No. 6: (**a**,**b**) low magnification; (**c**) high magnification.

**Figure 8:**
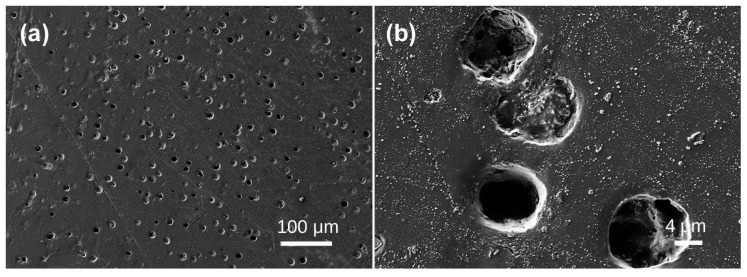
SEM images of Baltic amber sample No. 7: (**a**) low magnification; (**b**) high magnification.

**Figure 9:**
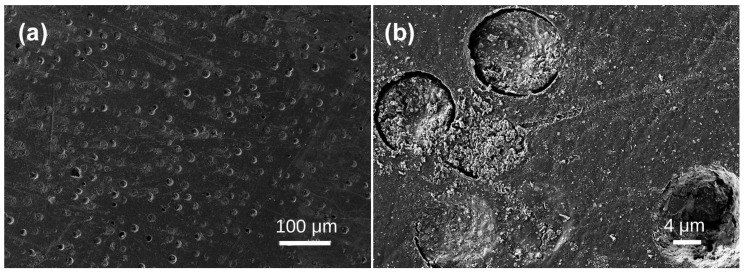
SEM images of Baltic amber sample No. 8: (**a**) low magnification; (**b**) high magnification.

The test area of Sample 6 is situated within an opaque white region characterized by flow marks and a dense clustering of bubbles. This density results in the intersection and coalescence of adjacent bubbles, creating an irregularly sized bubble. The predominant bubble diameter was 10 μm, with a limited number measuring 4–5 μm (Figure 7a). An extraordinarily big bubble measuring 50 μm was observed (Figure 7b). At 1760× magnification, hollow, curving tubules were discernible at the bubble (Figure 7c), displaying a crack-like distribution pattern. The interior surfaces of these cylindrical tubes were polished and oriented vertically. Sample 7 was examined in four segments. In comparison to Samples 1–5, it displayed a markedly greater number of bubbles, which were densely and irregularly dispersed across the matrix. The bubbles exhibited little deformation, resembling spherical bubbles predominantly in two size classifications: the principal bubbles measured 10 μm in diameter, while a lesser number ranged from 4 μm to 5 μm (Figure 8). Sample 8 displays a multitude of shallow and deep bubbles spread irregularly. Oriented stress fractures are evident surrounding the bubbles. bubble sizes predominantly measure 10 μm in diameter, with the maximum reaching 15 μm and the minimum at 5 μm (Figure 9).

**Figure 10:**
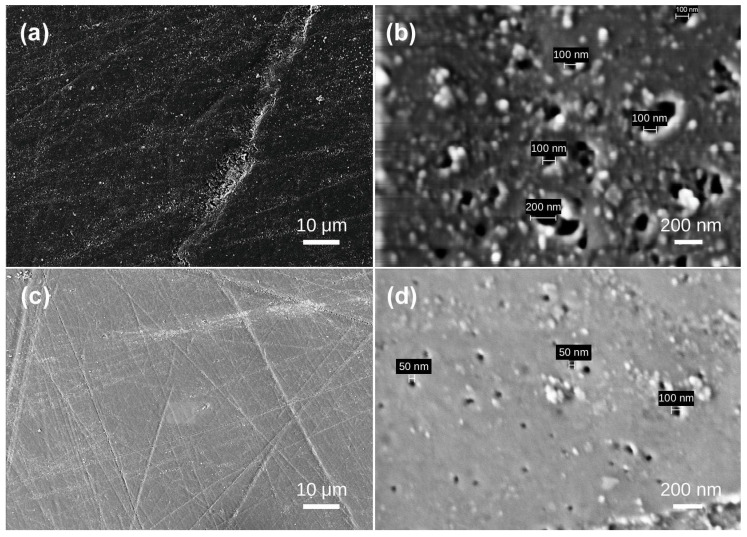
SEM images of Baltic amber sample No. 10: (**a**,**c**) low magnification; (**b**,**d**) high magnification.

Sample 10 has a multitude of highly dense bubbles, characterized by minuscule bubble diameters. The bubble sizes of Samples 1 to 8 are quantified in micrometers, whereas those of Sample 9 are quantified in nanometers. At a magnification of 35,600×, the principal bubble diameter is roughly 100 nm, with a maximum diameter of 200 nm (Figure 10a) and a minimum diameter of approximately 50 nm (Figure 10b).

### 3.2. FTIR and UV Analysis

**Figure 11:**
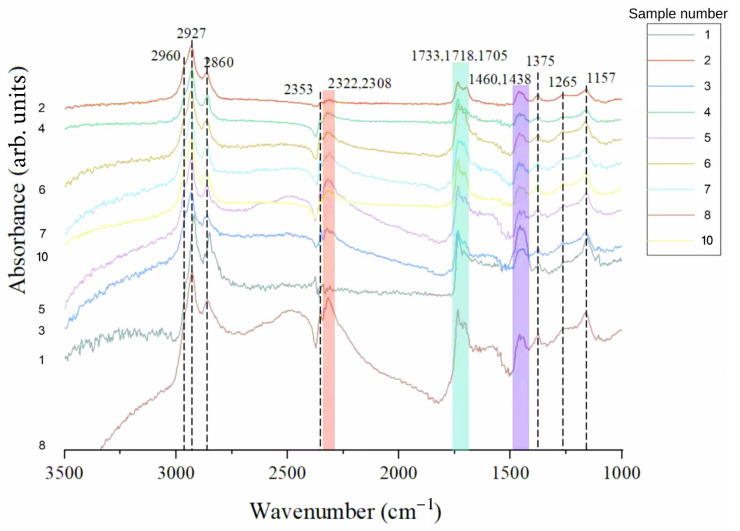
FTIR spectra are shown for nine samples, as one specimen was excluded due to damage.

FTIR spectroscopy was utilized on all samples to assess the influence of chemical composition on the observed color variation (Figure 11). The predominant absorption characteristics are uniform across all specimens: C–H stretching vibrations are observed in the 3000–2800 cm^−1^ region, with bands at ~2927 and ~2860 cm^−1^ attributed to methylene (–CH_2_–) groups, and a band near ~2960 cm^−1^ assigned to the antisymmetric stretching vibration of methyl (–CH_3_) groups; the symmetric stretching vibration of methyl groups (~2870 cm^−1^) may overlap with the methylene band; an ester carbonyl (C=O) stretching band is located at approximately 1733 cm^−1^; aliphatic C–H deformation modes are detected in the 1460–1375 cm^−1^ range; and C–O–C stretching absorptions are evident at 1265 and 1157 cm^−1^. This collection of functional groups aligns entirely with the succinite-type resin system typical of Baltic amber, as demonstrated in earlier spectroscopic investigations. In addition, two characteristic absorption regions are highlighted in Figure 11, including the carbonyl (C=O) stretching band near ~1730 cm^−1^ and the C–O–C stretching vibrations in the 1200–1300 cm^−1^ range. These features are consistently observed across all samples and further support their compositional similarity. The absorption bands observed between 2308 and 2353 cm^−1^ in many spectra are ascribed to ambient CO_2_ and provide no insights into the sample composition.

Sample 8 exhibits a slight yet significant deviation: its carbonyl band is broader than those of the other samples and features a shoulder near 1705 cm^−1^, indicating that some carbonyl groups are situated in a hydrogen-bonded or weakly conjugated chemical environment, potentially indicative of localized oxidative modification. This property, however, exhibits no systematic correlation with the observable color variations among samples, and its impact on absorption within the visible spectral range is anticipated to be minimal.

The spectroscopic consistency among samples exhibiting a complete spectrum of visual characteristics from yellow to white indicates that differences in molecular composition and inherent chromophore content are probably not the primary determinants of color. These results underscore the significance of microstructural impacts.

From an optical standpoint, internal gas inclusions may function as scattering centers. When the size of internal bubbles is comparable to or larger than the wavelength of visible light, scattering is dominated by Mie-type interactions. In this regime, scattering remains significant over a broad spectral range and exhibits relatively weak wavelength dependence, resulting in diminished transmission and amplified diffuse reflection. The aggregate impact of these scattering events can yield the observed white, non-selective optical response in these materials. The combined evidence of spectroscopic uniformity and microstructural variation indicates that the color variations in Baltic amber are mostly influenced by light scattering from internal bubbles, rather than significant variances in chemical composition.

**Figure 12:**
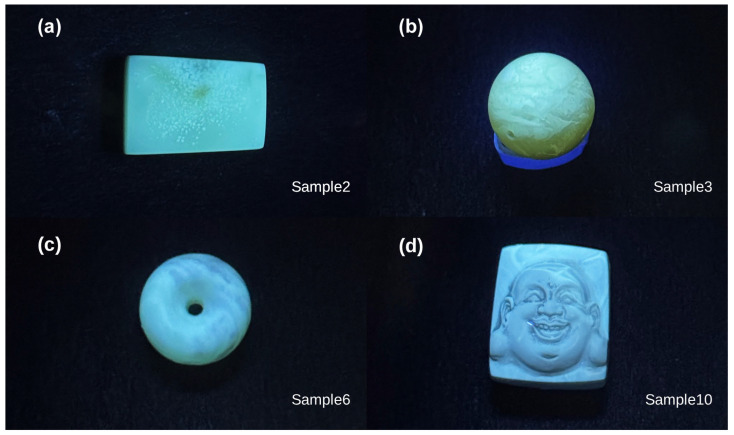
Representative photographs of selected Baltic amber samples (Samples 2, 3, 6, and 10) under long-wave UV illumination, showing similar blue–violet fluorescence. Images are presented for qualitative comparison.

In addition, qualitative visual observations under ultraviolet (UV) illumination indicate that the samples exhibit broadly similar fluorescence appearance across different appearance types. As shown in Figure 12, the selected samples (Samples 2, 3, 6, and 10) display a visually observed blue–violet fluorescence under long-wave UV illumination. It should be noted that these observations are qualitative in nature and are not based on quantitative spectroscopic measurements. Therefore, they provide only limited supplementary evidence and should not be considered a primary basis for compositional interpretation [13].

The apparent variation in fluorescence intensity is based on qualitative visual observation only and is not quantitatively evaluated. Overall, the similarity in UV response among samples, together with the consistent FTIR results, indicates that chemical composition alone is unlikely to account for the observed differences. Instead, these observations further support the interpretation that physical factors, particularly bubble-induced scattering, play a dominant role in determining the optical appearance [14].

## 4. Discussion

### 4.1. Statistical Analysis of Internal Bubble Characteristics in Baltic Amber

This research selected ten Baltic amber specimens varying in hue from yellow to white. A comprehensive examination of a particular internal region of each sample was performed using scanning electron microscopy (SEM). In this study, structural characterization was conducted primarily using SEM to examine the microstructural features of bubbles within the amber samples. FTIR spectroscopy was performed to evaluate the functional group characteristics of the samples, while no additional spectroscopic techniques (e.g., Raman or NMR) were employed, as the focus of this work is on the relationship between bubble microstructure and optical behavior.

The bubble size, number, and density characteristics of beeswax-amber in various colors were statistically evaluated, with the results displayed in Table 2.

Microscopic analysis of Samples 1 and 2 showed no observed bubbles, signifying the uniform condition of these beeswax-amber specimens. In the absence of bubble interference, the beeswax-amber displays its intrinsic matrix hue. Upon examining Samples 3 and 4, a negligible number of bubbles with an exceedingly low area ratio is apparent, and their hue stays largely unaltered by the presence of bubbles.

Sample 5, the darkest beeswax-amber among all specimens, displays significantly distinct bubble properties in contrast to the other four samples. The number of bubbles in the studied area has markedly risen, along with an increase in their proportion. Despite comprising about 0.186% of the overall area, bubbles of diverse sizes are dispersed across the viewing zone.

Samples 1 to 5 exhibit golden beeswax-amber in diverse spectral contributions. Samples 6, 7, and 8, however, display beeswax-amber with a white base spectral contribution, showcasing various spectral contributions of warm white. These three samples exhibit a substantial rise in bubble number and density, resulting in the formation of many bubble clusters. Sample 6 displays the most luminous white of the three, with a bubble area ratio of 5.431%. It also contains the most densely packed and abundant bubbles in comparison. As bubble size, number, and density (e.g., Samples 6–8), the frequency of light scattering events within the matrix increases, leading to enhanced Mie scattering. The color disparities between white and yellow beeswax-amber, together with the analysis of bubble number and density, suggest that bubbles are a contributing factor to the hue of white beeswax-amber. The increased number and density of bubbles enhance the intensity of the beeswax-amber’s white tint.

Sample 10, the lightest sample among all specimens and characterized by a greater contribution in the blue range of the visible spectrum, exhibits internal characteristics markedly different from the others. Microscopic observation required significantly higher magnification than for other samples. Under these conditions, irregularly shaped bubbles became faintly discernible within the sample. The field of view narrowed considerably, yet the bubble area ratio remained at 2.291%, indicating extremely dense bubble distribution. The most significant difference compared to other samples lies in bubble size. While bubbles in other samples measure micrometers in diameter, those in Sample 10 are now measured in nanometers, with most averaging approximately 100 nm. Concurrently, bubble number and density have substantially increased. These characteristic changes invariably influence the coloration of white beeswax-amber.

In summary, the bubble size, number, and density exhibit distinct variations across differently colored samples. Samples 1–4 are beeswax-amber with pronounced yellow spectral contributions, containing very few internal bubbles. Their bubble characteristics are insufficient to structurally alter the beeswax-amber’s coloration, so the primary hue remains the beeswax-amber’s intrinsic body color. In contrast, Samples 6–8, and 10 represent white beeswax-amber with varying hues. These samples exhibit a dramatic increase in bubble number and extremely dense distribution, displaying microstructural characteristics markedly different from the yellow samples. This indicates a close correlation between beeswax-amber coloration and internal bubble characteristics. Although Sample 5 exhibits a noticeable number of bubbles, the overall bubble area ratio remains relatively low. The sample maintains a rich yellow hue macroscopically, suggesting that the bubble number and density have not yet reached a threshold level to significantly influence the beeswax-amber’s coloration. The hue of amber is determined by its microstructure, chemical makeup, and potential contaminants. Prior research indicates that chromophoric groups, including conjugated double bonds and carbonyl (C=O) groups, along with trace elements (e.g., Fe, Mn), influence light absorption and contribute to color variation [15]. This study primarily examines the influence of bubble microstructures on light scattering and optical characteristics. The size, number, and geographical distribution of bubbles within the amber matrix were quantitatively described using image analysis. These structural characteristics offer a foundation for assessing their possible impact on optical behavior. The likelihood of light interacting with these interior features rises with the size and density of bubbles. This implies that light propagation within the material is modulated by bubble microstructure.

### 4.2. Formation Mechanism of White Baltic Beeswax-Amber

This study demonstrates that the hue of beeswax-amber is affected by the size, number, and density of internal bubbles, offering significant patterns for the examination of beeswax-amber’s coloration. Bubble properties significantly influence the coloring of white beeswax-amber. Smaller bubble diameters, larger quantities, and denser distributions yield a purer white spectral contribution. This phenomenon entails an analysis of the principles that dictate the hue of white beeswax-amber.

Speculation about the intrinsic origin of white beeswax-amber’s hue is linked to the same phenomena of white streaks. When mineral particles like garnet, beryl, or pyroxene are reduced to a sufficiently small size, they lose their inherent hue and transform into pale or white, powdery substances. For instance, garnet displays a white streak color. Beryl displays a pale or nearly white streak hue. This phenomenon is acknowledged; however, its precise cause remains unidentified.

The hue of these small particles in the streak phenomena closely resembles that of nanoscale bubbles within white beeswax-amber. Both have exceptionally fine proportions and are perceptibly white to the unaided eye. Nonetheless, they differ fundamentally: the refractive index of the mineral particles in white streaks surpasses that of air, but the bubbles inside beeswax-amber possess a refractive index inferior to that of the beeswax-amber matrix itself. Both structures demonstrate considerable discrepancies in refractive index compared to their surrounding medium. The disparity in refractive index is a critical determinant of scattering intensity. When light interacts with multiple little particles, scattering transpires. Fine particles and thick bubbles are critical for scattering, resulting in the dispersion of light waves in all directions and creating a uniform scattering effect. The streak phenomena and the scattering from bubbles in beeswax-amber display powerful, uniform scattering with a white hue, indicating a relationship to Mie scattering.

Mie scattering is a classical electromagnetic scattering theory introduced by German scientist Gustav Mie in 1908 [16], which elucidates the events occurring when planar electromagnetic waves interact with homogenous spherical particles. Mie scattering necessitates scattering particles with diameters equivalent to the wavelength of the incident light, a sufficient number of uniformly distributed particles within the media, and a substantial refractive index disparity between the particles and the surrounding medium [17]. The internal composition of beeswax-amber precisely satisfies the rigorous criteria for Mie scattering. This investigation examines samples 6 to 8, and 10 of white beeswax-amber, revealing the presence of interior bubbles with in diameter between 0.1 and 10 μm. The scatterer in the visible light spectrum (about 400–700 nm) range in size from tens of nanometers to several micrometers. Consequently, the interior bubble diameters are analogous to the dimension of the scatterer necessary for Mie scattering. The scanning electron microscope images of the samples distinctly demonstrate consistently and densely dispersed bubbles within the white beeswax-amber. A refractive index contrast exists between the gas-filled bubbles (n ≈ 1.0) and the surrounding resin matrix, whose refractive index is typically reported to range from approximately 1.53 to 1.55 [18]. Mie scattering is a significant phenomenon affecting the colors of beeswax-amber, offering a macroscopic optical foundation for the differences in amber’s transparency and saturation [19]. When light traverses a heterogeneous medium, the boundary conditions of its electromagnetic field alter, resulting in many optical phenomena, including reflection, refraction, and scattering [20].

The trajectory of light is affected by the size, number, and density of scattering particles, namely internal bubbles within beeswax-amber. As the disparity in refractive indices between these particles and the surrounding medium escalates, the effects of reflection and refraction at the interface markedly strengthen. The increase in bubble size, number, and density enhances the scattering cross-section, thereby strengthening multiple scattering within the amber matrix. As scattering events become more frequent, the propagation of light shifts from predominantly linear transmission to a more randomized and diffusive regime [21].

As a result, the role of direct transmission is markedly diminished, but diffuse reflectance becomes increasingly prevalent. The shift from absorption-dominated to scattering-dominated optical properties causes a reduction in transmittance and a concomitant decline in color saturation, finally producing a milky white look.

The optical characteristics of Baltic amber are influenced by many causes. Within the scope of this study, the observed color variations are primarily attributed to differences in bubble microstructures, which regulate the intensity of Mie scattering. This phenomenon occurs when light experiences numerous scattering and mixing, hence extending and elongating its propagation routes within materials. Partial light experiences numerous scattering during propagation, resulting in things appearing milky white [19,20]. Variations in bubble size, number, and density therefore play a critical role in controlling the degree of whiteness in beeswax amber.

### 4.3. Mechanism of Color Formation in Baltic Beeswax-Amber

The color transition from yellow to white in Baltic amber reflects systematic variations in interior bubble structures, as indicated by the previously discussed microstructural analysis results and conclusions. This suggests that optical phenomena resulting from the beeswax-amber’s microstructure are essential in color representation. Bubble size, number, and density are critical parameters affecting colors, which is fundamentally governed by optical effects and internal microstructure. Beeswax-amber mostly consists of a terpene resin polymer. Previous studies have shown that during geological fossilization and oxidation processes, molecular chains in amber may generate chromophoric groups, including conjugated double bonds and carbonyl (C=O) groups. Additionally, trace metal ions such as iron (Fe) and manganese (Mn) from the depositional environment may permeate the resin, thereby enhancing light absorption at specific wavelengths [15]. These factors are considered to play an important role in determining the intrinsic coloration of amber, particularly for hues ranging from yellow and orange-yellow to deep orange-red. Reference Consequently, specimens with few bubbles (e.g., Specimens 1–4) display body colors primarily influenced by the selective absorption of visible light by the beeswax-amber matrix, resulting in intensely saturated beeswax-amber spectral contributions at the macroscopic scale.

As the quantity and spatial proportion of bubbles augment (e.g., Samples 6, 7, 8), light scattering within the matrix bubbles escalates, resulting in Mie scattering. When bubble density reaches a sufficient level, incident light undergoes simultaneous scattering, absorption, and transmission within the amber matrix. As scattering intensity increases with bubble concentration, the proportion of light transmitted through the specimen decreases correspondingly, while scattered light is redirected diffusely in all directions. The net effect is a suppression of transmitted intensity and a dominance of diffuse reflection over selective absorption, producing the milky, opaque appearance characteristic of white beeswax amber [22]. Concurrently, the foundational hue of the beeswax-amber matrix merges with the milky white produced by scattering, yielding warm white beeswax-amber with various intensities of color at the macroscopic scale. Baltic amber possesses a significant concentration of succinic acid, indicative of its distinctive resin stability. The emergence of bubbles is linked to various volatile constituents, such terpenes and succinic acid [23], This promotes the conservation of interior microstructures, hence augmenting light scattering effects indirectly. Moreover, reduced chromophore ion concentration and diminished oxidation levels effectively inhibit selective light absorption, leading to an optical impact mostly governed by scattering.

White beeswax-amber displays variations in spectral characteristics, corresponding to differences in the detected light. In samples where the detected light has a greater contribution in the blue range of the visible spectrum (e.g., Sample 10), the matrix’s intrinsic yellow base hue is largely suppressed, suggesting that this beeswax-amber variety is not exclusively influenced by Mie scattering but arises from other interacting causes. The detected variations are presumably due to Rayleigh scattering. Beeswax-amber encompasses bubbles ranging from nanometer to micrometer dimensions, wherein varying scatterer sizes establish unique scattering systems. Nanoscale bubble gaps demonstrate exceptional light scattering efficiency, facilitating Rayleigh scattering in these circumstances. Short-wavelength light (blue-violet) is preferentially dispersed [24], resulting in a higher contribution in the blue range of the visible spectrum, exemplified by Sample 10. Mie scattering prevails when scatterer sizes correspond with visible light wavelengths, enabling the intrinsic body color of beeswax-amber to affect the detected light, resulting in a greater contribution in the yellow–orange range of the visible spectrum (e.g., Sample 8). The variation in spectral characteristics within white beeswax-amber may arise from the synergistic effects of Mie scattering and Rayleigh scattering. Full-spectrum visible light experiences numerous diffuse reflections, obscuring the matrix’s intrinsic pale yellow hue. The ultimate color is probably contingent upon the interplay of scatterer at various scales.

Research suggests that the color shift from yellow to white in Baltic amber is influenced by multiple chemical factors. The yellow coloration of beeswax-amber is mainly influenced by selective absorption within its matrix, while white beeswax-amber is characterized by light scattering effects caused by internal bubbles. This color modification basically signifies a change in the predominance of absorption and scattering optical mechanisms. This work seeks to identify patterns in beeswax-amber colors by quantitatively analyzing internal feature scales and coloration, providing a systematic optical explanation for the varied hues of Baltic amber.

## 5. Conclusions

The color of Baltic amber is inextricably linked to its internal bubble characteristics. A minor amount of bubbles cannot influence the coloration of beeswax-amber. When the size, quantity, and density of bubbles exceed a certain threshold, white beeswax-amber displaying various hues is produced. The color of beeswax-amber depends on whether selective absorption or light scattering predominates. The divergence between these two visual systems produces distinct macroscopic colors. The microscopic bubble architecture of white beeswax-amber conforms to the principles of Mie scattering, as certain specimens include several nanoscale bubbles. The distribution of particles of varying sizes may produce a synergistic effect of Rayleigh and Mie scattering, resulting in a white appearance with a greater contribution from the blue region of the visible spectrum.

## Figures and Tables

**Table 1:** Color and morphology of Baltic amber samples.

Sample No.	Color (Munsell Notation)	Shape	Morphological Description
1	Yellow-green (bright yG)	Freeform	Brown mottled textures in the center; compact texture
2	Yellow (bright Y)	Rectangular cabochon	Flow structures present; porous texture
3	Orange-yellow (intense oY)	Spherical	Flow structures present; compact texture
4	Orange (vivid O)	Spherical	Brown mottled textures; compact texture
5	Orange-red (deep oR)	Rectangular cabochon	Compact texture
6	White (V 8)	Abacus bead	Transparent–opaque flow structures; compact texture
7	White (V 8)	Abacus bead	Flow structures present; compact texture
8	White (V 7)	Abacus bead	Flow structures present; compact texture
9	Yellow-green (bright yG)	Irregular	Yellowish-brown rind; compact texture
10	White (V 9)	Carved piece	Compact texture

**Table 2:** Statistical characteristics of bubbles in Baltic amber samples.

Sample No.	SEM Magnification	Number of Bubbles	Bubble Diameter (μm)	Area Fraction (%)
1	106	0	-	-
2	127	0	-	-
3	129	2	15	0.006
4	1090	2	3	0.105
5	120	60	5–10	0.186
6	208	678	4–10	5.431
7	217	314	10	2.638
8	154	325	10–15	2.173
9	n.d.	n.d.	n.d.	n.d.
10	35,660	25	0.1–0.2	2.291

“-“ indicates not applicable (no bubbles observed); “n.d.” indicates no data available.

## Data Availability

The original contributions presented in this study are included in the article. Further inquiries can be directed to the corresponding author.
